# Supplementary material for: Circulating Interleukin-8 Dynamics Parallels Disease Course and Is Linked to Clinical Outcomes in Severe COVID-19
Source: Viruses. 2023 Feb 16;15(2):549. doi: 10.3390/v15020549 (PMC9960352; doi:10.3390/v15020549)
Supplement: Supplementary file 1 [file viruses-15-00549-s001.zip › viruses-2038220-supplementary.pdf]

Circulating interleukin-8 dynamics parallels disease course and is linked to clinical outcomes in severe COVID-19

Ranit D’Rozario et al.

Supplemental information:

Supplemental figure legends

Supplemental figure 1-4

Supplemental table 1-7

Supplemental codes 1-6

Supplemental figure legends:

Supplemental figure 1. Plasma Eotaxin and IFNγ concentrations and clinical outcomes in severe COVID-19 patients. A) Receiver operator characteristic curve (black line) of plasma levels of Eotaxin to derive remission versus non-remission (death), the marked point denotes the specificity and sensitivity with the derived cut-off. **B)** Receiver operator characteristic curve (black line) of plasma levels of IFNγ to derive remission versus non-remission (death), the marked point denotes the specificity and sensitivity with the derived cut-off. **C)** Survival of patients till day 30 post-enrolment are compared in a Kaplan-Meier curve between patients with plasma level of Eotaxin below ($Eotaxin_{lo}$, black line) or above ($Eotaxin_{hi}$, red line) the cut-off derived from the ROC curve shown in (A). Surviving patients were censored on day 30 post-enrolment. **D)** Survival of patients till day 30 post-enrolment are compared in a Kaplan-Meier curve between patients with plasma level of IFNγ below ($IFNG_{lo}$, black line) or above ($IFNG_{hi}$, red line) the cut-off derived from the ROC curve shown in (B). Surviving patients were censored on day 30 post-enrolment. **E)** Hospital stay duration (time to remission) of the patients from both groups, $Eotaxin_{lo}$ (black line) and $Eotaxin_{hi}$ (red line), since the day of enrolment are plotted in an ascending Kaplan-Meier curve. Deaths and non-remission at day 30 post-enrolment were censored. **F)** Hospital stay duration (time to remission) of the patients from both groups, $IFNG_{lo}$ (black line) and $IFNG_{hi}$ (red line), since the day of enrolment are plotted in an ascending Kaplan-Meier curve. Deaths and non-remission at day 30 post-enrolment were censored. For the outcome comparisons shown in (C) - (F) Mantel Cox log rank test was performed. Number of patients at risk on different days and the Mantel Haenszel Hazard Ratio is shown.

Supplemental figure 2. Comparison of viral load and neutralizing antibody content of plasma among $IL8_{lo}$ and $IL8_{hi}$ patients. A) Scatter plot showing CT values derived from RT-PCR for SARS-CoV-2 from nasopharyngeal swabs, comparing $IL8_{lo}$ and $IL8_{hi}$ patients. **B)** Scatter plot showing neutralizing antibody content of plasma derived from a surrogate virus neutralization assay, comparing $IL8_{lo}$ and $IL8_{hi}$ patients. In both cases two tailed Kolmogorov-Smirnof test was performed.

Supplemental figure 3. Clinical outcome comparison between patient subgroups classified based on mean cut-off value for plasma IL-8 level over three time-points. A) Survival of patients till day 30 post-enrolment are compared in a Kaplan-Meier curve between patients with plasma level of IL-8 below ($IL8_{lo}$, black line) or above ($IL8_{hi}$, red line) the mean cut-off value from three time-points. Surviving patients were censored on day 30 post-enrolment. **B)** Hospital stay duration (time to remission) of the patients from both groups, $IL8_{lo}$ (black line) and $IL8_{hi}$ (red line), since the day of enrolment are plotted in an ascending Kaplan-Meier curve. Deaths and non-remission at day 30 post-enrolment were censored. For the outcome comparisons shown Mantel Cox log rank test was

performed. Number of patients at risk on different days and the Mantel Haenszel Hazard Ratio is shown.

Supplemental figure 4. Effect of age, gender and co-morbidities with circulating IL-8 in severe COVID-19 patients. A) Correlation of plasma IL-8 level on the day of enrolment with age of the severe COVID-19 patients. **B)** Comparison of plasma IL-8 level on the day of enrolment among males and females. **C)** Comparison of plasma IL-8 level on the day of enrolment among severe COVID-19 patients with or without the major comorbidities, viz. type 2 diabetes mellitus (T2DM) and hypertension (HTN).

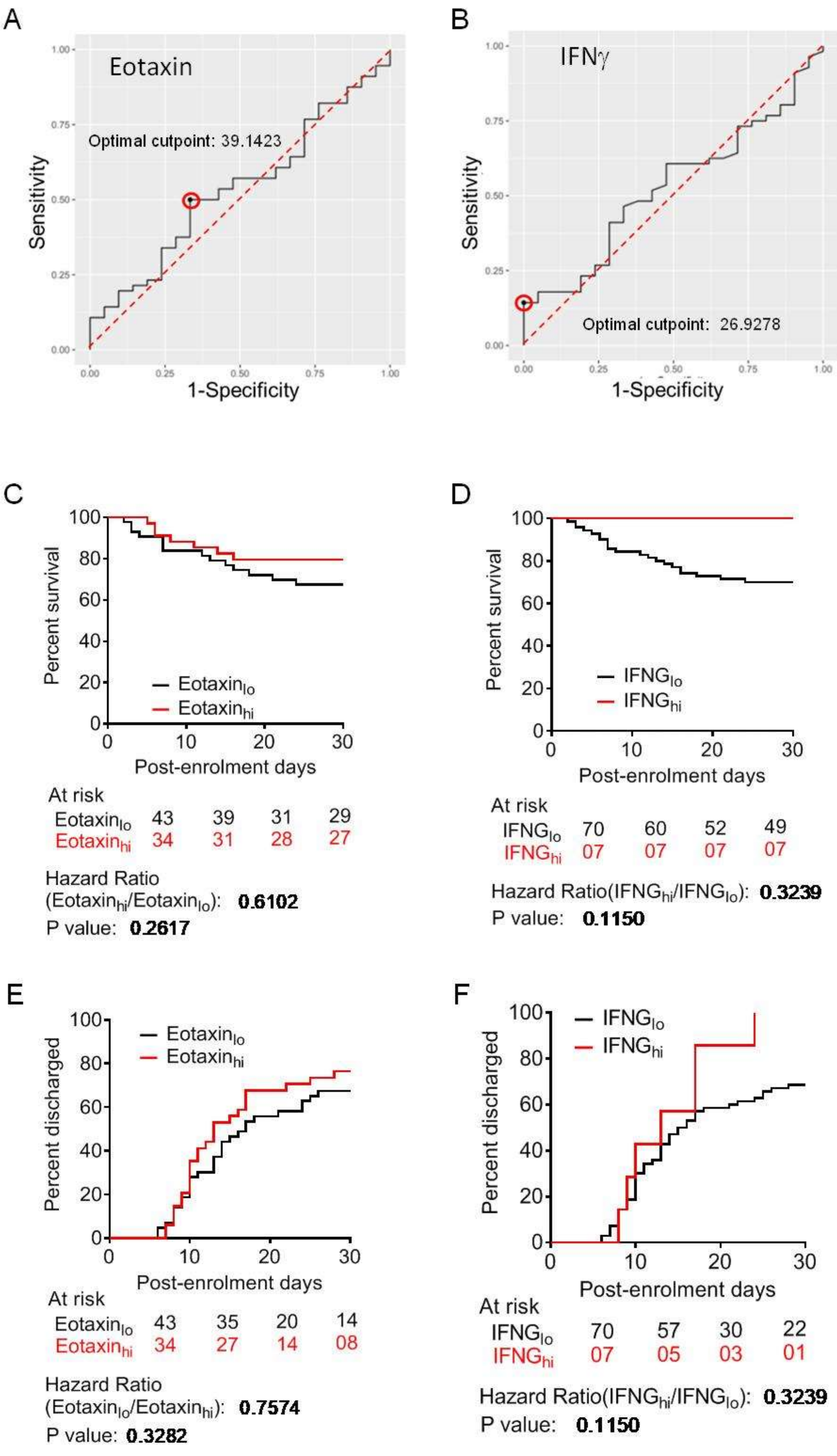

Supplemental figure 1

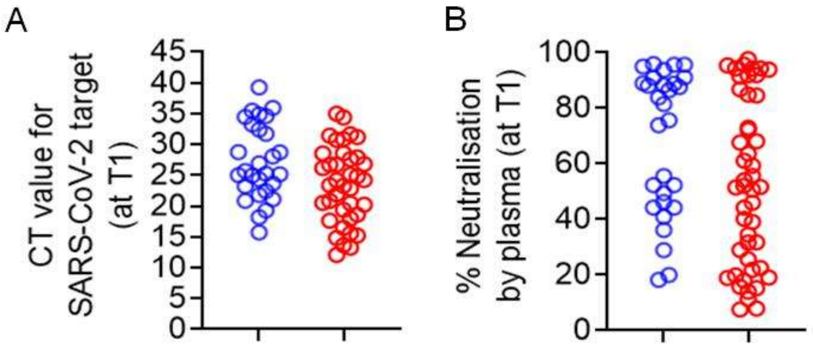

Supplemental figure 2

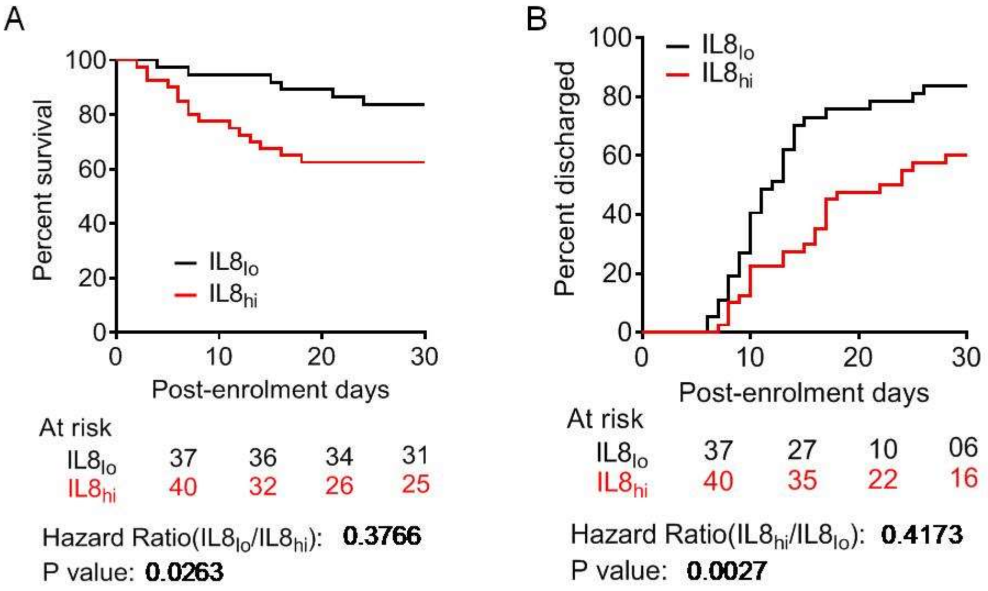

Supplemental figure 3

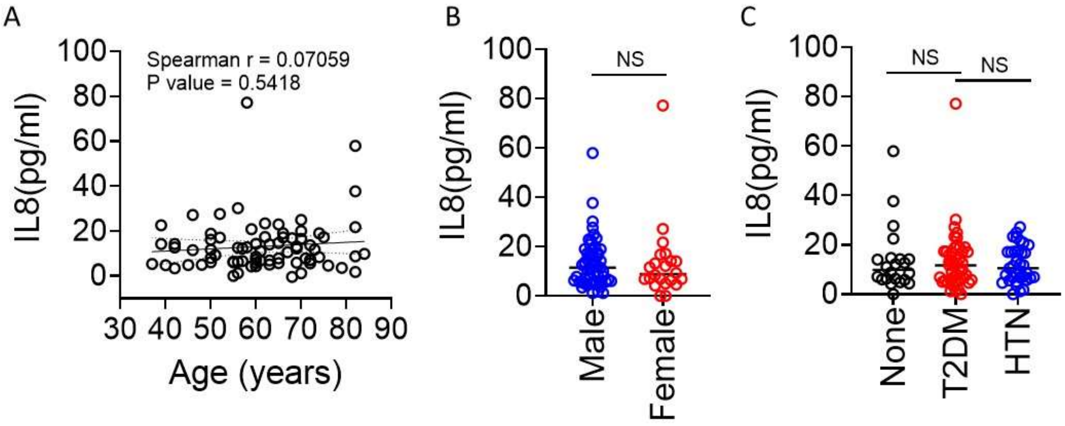

Supplemental figure 4

Supplemental table 1. Cohort characteristics.

	All ARDS	Low IL8	High IL8
**Demography**			
Male	N=55, 71.43%, Age=62±12.21years	N=20, 25.97%, Age=60±12.59years	N=35, 45.45%, Age=62±12.03years
Female	N=22, 28.57%, Age=60±10.54years	N=10, 12.99%, Age=60.5±8.43years	N=12, 15.58%, Age=60.5±12.36years
**Major co-morbidities**			
Type 2 diabetes	N=46, 59.74%	N=18, 23.38%	N=28, 36.36%
Hypertension	N=35, 45.45%	N=13, 16.88%	N=22, 28.57%
**Pharmacotherapy**			
Standard-of-care	N=77, 100%	N=30, 100%	N=47, 100%
Remdesivir	N=25, 32.46%	N=10, 12.99%	N=15, 19.48%
Convalescent plasma	N=38, 49.35%	N=14, 18.18%	N=24, 31.17%

Supplemental table 2. Enriched pathways for upregulated genes in $IL8_{hi}$ at time-point 1 as compared to $IL8_{lo}$ at the same time-point.

Cluster ID	List of pathway	Total	Expected	Hits	P.Value	FDR
ECM Biosynthesis pathway	Chondroitin sulfate biosynthesis	23	1.14	5	0.00461	0.528
	Chondroitin sulfate/dermatan sulfate metabolism	53	2.62	6	0.0453	0.784
	Keratan sulfate biosynthesis	27	1.33	5	0.0094	0.528
	Glycosaminoglycan metabolism	118	5.83	12	0.0133	0.528
	MPS VI - Maroteaux-Lamy syndrome	118	5.83	12	0.0133	0.528
	Mucopolysaccharidoses	118	5.83	12	0.0133	0.528
	MPS IX - Natowicz syndrome	118	5.83	12	0.0133	0.528
	MPS IIIB - Sanfilippo syndrome B	118	5.83	12	0.0133	0.528
	MPS I - Hurler syndrome	118	5.83	12	0.0133	0.528
	MPS II - Hunter syndrome	118	5.83	12	0.0133	0.528
	MPS VII - Sly syndrome	118	5.83	12	0.0133	0.528
	MPS IV - Morquio syndrome A	118	5.83	12	0.0133	0.528
	MPS IIIC - Sanfilippo syndrome C	118	5.83	12	0.0133	0.528
	MPS IV - Morquio syndrome B	118	5.83	12	0.0133	0.528
	MPS IIIA - Sanfilippo syndrome A	118	5.83	12	0.0133	0.528
	MPS IIID - Sanfilippo syndrome D	118	5.83	12	0.0133	0.528
	Keratan sulfate/keratin metabolism	32	1.58	5	0.0192	0.584
	Galactose catabolism	5	0.247	2	0.022	0.585
	Metabolism of carbohydrates	258	12.8	21	0.0162	0.555
Transcription of HIV genome	Transcription of the HIV genome	64	3.16	8	0.0129	0.528
	Tat-mediated elongation of the HIV-1 transcript	45	2.22	6	0.0223	0.585
	Formation of HIV-1 elongation complex containing HIV-1 Tat	45	2.22	6	0.0223	0.585
	HIV-1 Transcription Elongation	45	2.22	6	0.0223	0.585

	Formation of HIV-1 elongation complex in the absence of HIV-1 Tat	46	2.27	6	0.0246	0.585
	Late Phase of HIV Life Cycle	108	5.34	10	0.0402	0.772
	Formation of the HIV-1 Early Elongation Complex	34	1.68	6	0.00579	0.528
	HIV-1 Transcription Initiation	41	2.03	6	0.0145	0.528
	RNA Polymerase II HIV-1 Promoter Escape	41	2.03	6	0.0145	0.528
	Abortive elongation of HIV-1 transcript in the absence of Tat	23	1.14	4	0.0246	0.585
Unfolded protein response	Unfolded Protein Response	66	3.26	10	0.00135	0.528
	Activation of Chaperone Genes by XBP1(S)	46	2.27	6	0.0246	0.585
	Activation of Chaperones by IRE1alpha	49	2.42	6	0.0325	0.662
NOTCH signaling	NOTCH1 Intracellular Domain Regulates Transcription	50	2.47	6	0.0354	0.709
	Signaling by NOTCH	95	4.7	9	0.0443	0.777
Cytokine signaling	Cytokine Signaling in Immune system	286	14.1	22	0.0253	0.59
	Immune System	1140	56.2	68	0.0479	0.79
	Interferon alpha/beta signaling	68	3.36	9	0.0059	0.528
	Interferon Signaling	173	8.55	15	0.0237	0.585
	Antigen Presentation: Folding, assembly and peptide loading of class I MHC	25	1.24	4	0.0326	0.662
Toll like receptor cascade	TRIF-mediated TLR3/TLR4 signaling	87	4.3	9	0.0272	0.617
	MyD88-independent cascade	88	4.35	9	0.029	0.617
	Toll Like Receptor 3 (TLR3) Cascade	88	4.35	9	0.029	0.617
	Toll-Like Receptors Cascades	123	6.08	11	0.0402	0.772

	NF-kB activation through FADD/RIP-1 pathway mediated by caspase-8 and -10	12	0.593	3	0.0189	0.584
	TRAF6 mediated NF-kB activation	16	0.791	3	0.0415	0.774
	Activation of NIMA Kinases NEK9, NEK6, NEK7	7	0.346	2	0.0434	0.774
	RIG-I/MDA5 mediated induction of IFN-alpha/beta pathways	67	3.31	7	0.0463	0.784
Semaphorin interactions	Semaphorin interactions	72	3.56	12	0.000183	0.257
	Sema4D induced cell migration and growth-cone collapse	29	1.43	6	0.00252	0.528
	Sema4D in semaphorinsignaling	34	1.68	6	0.00579	0.528
	SEMA3A-Plexin repulsion signaling by inhibiting Integrin adhesion	14	0.692	3	0.029	0.617
Transcriptional regulation	RNA Pol II CTD phosphorylation and interaction with CE	28	1.38	4	0.047	0.784
	RNA Pol II CTD phosphorylation and interaction with CE	28	1.38	4	0.047	0.784
	RNA Polymerase II Pre-transcription Events	62	3.06	8	0.0107	0.528
	RNA Polymerase II Transcription Elongation	46	2.27	6	0.0246	0.585
	Formation of RNA Pol II elongation complex	46	2.27	6	0.0246	0.585
	Formation of the Early Elongation Complex	34	1.68	6	0.00579	0.528
	Transcription	149	7.36	15	0.00659	0.528
	RNA Polymerase II Promoter Escape	41	2.03	6	0.0145	0.528
	RNA Polymerase II Transcription Pre-Initiation And Promoter Opening	41	2.03	6	0.0145	0.528

	RNA Polymerase II Transcription Initiation	41	2.03	6	0.0145	0.528
	RNA Polymerase II Transcription Initiation And Promoter Clearance	41	2.03	6	0.0145	0.528
	RNA Polymerase II Transcription	107	5.29	11	0.0162	0.555
	Regulation of Cholesterol Biosynthesis by SREBP (SREBF)	39	1.93	5	0.0415	0.774
	mRNA Processing	140	6.92	12	0.0436	0.774

Supplemental table 3. Enriched pathways for downregulated genes in $IL8_{hi}$ at time-point 1 as compared to $IL8_{lo}$ at the same time-point.

Cluster ID	List of pathway	Total	Expected	Hits	P.Value	FDR
Transcription of HIV genome	HIV Infection	214	19.5	33	0.00169	0.157
	HIV-1 Transcription Initiation	41	3.73	10	0.00303	0.157
	RNA Polymerase II HIV-1 Promoter Escape	41	3.73	10	0.00303	0.157
	Late Phase of HIV Life Cycle	108	9.83	23	8.26E-05	0.0289
	HIV Life Cycle	128	11.7	24	0.000454	0.0844
	Transcription of the HIV genome	64	5.83	15	0.000482	0.0844
	Formation of HIV-1 elongation complex in the absence of HIV-1 Tat	46	4.19	10	0.00728	0.213
	Tat-mediated elongation of the HIV-1 transcript	45	4.1	9	0.0183	0.297
	Formation of HIV-1 elongation complex containing HIV-1 Tat	45	4.1	9	0.0183	0.297
	HIV-1 Transcription Elongation	45	4.1	9	0.0183	0.297
	HIV-1 elongation arrest and recovery	33	3	7	0.0264	0.304
	Pausing and recovery of HIV-1 elongation	33	3	7	0.0264	0.304
Transcriptional regulation	RNA Pol II CTD phosphorylation and interaction with CE	28	2.55	7	0.0109	0.259
	RNA Pol II CTD phosphorylation and interaction with CE	28	2.55	7	0.0109	0.259
	Transcription	149	13.6	31	8.24E-06	0.00578
	RNA Polymerase II Transcription	107	9.74	22	0.000201	0.0565

	RNA Polymerase II Pre-transcription Events	62	5.65	14	0.00109	0.153
	RNA Polymerase I, RNA Polymerase III, and Mitochondrial Transcription	59	5.37	13	0.00207	0.157
	RNA Polymerase I Promoter Escape	22	2	7	0.00253	0.157
	RNA Polymerase II Promoter Escape	41	3.73	10	0.00303	0.157
	RNA Polymerase II Transcription Pre-Initiation And Promoter Opening	41	3.73	10	0.00303	0.157
	RNA Polymerase II Transcription Initiation	41	3.73	10	0.00303	0.157
	RNA Polymerase II Transcription Initiation And Promoter Clearance	41	3.73	10	0.00303	0.157
	RNA Polymerase I Transcription Initiation	23	2.09	7	0.00336	0.161
	RNA Polymerase I Promoter Clearance	24	2.19	7	0.00438	0.184
	RNA Polymerase I Transcription	26	2.37	7	0.00709	0.213
	Dual incision reaction in TC-NER	31	2.82	7	0.019	0.297
	Formation of transcription-coupled NER (TC-NER) repair complex	31	2.82	7	0.019	0.297
	RNA Polymerase I Chain Elongation	21	1.91	7	0.00187	0.157
	RNA Polymerase I Transcription Termination	23	2.09	7	0.00336	0.161
	RNA Polymerase II Transcription Elongation	46	4.19	10	0.00728	0.213
	Formation of RNA Pol II elongation complex	46	4.19	10	0.00728	0.213

	Transcription-coupled NER (TC-NER)	47	4.28	10	0.00851	0.226
	RNA Polymerase III Transcription	33	3	7	0.0264	0.304
	RNA Polymerase III Transcription Initiation	33	3	7	0.0264	0.304
	Elongation arrest and recovery	33	3	7	0.0264	0.304
DNA repair	DNA Repair	117	10.7	21	0.0018	0.157
	Resolution of AP sites via the multiple-nucleotide patch replacement pathway	17	1.55	5	0.0151	0.293
	Removal of DNA patch containing abasic residue	17	1.55	5	0.0151	0.293
	Nucleotide Excision Repair	53	4.83	10	0.0196	0.297
	Resolution of Abasic Sites (AP sites)	19	1.73	5	0.0244	0.304
	Base Excision Repair	19	1.73	5	0.0244	0.304
Host-virus interactions	Vpr-mediated nuclear import of PICs	32	2.91	8	0.00662	0.213
	Interactions of Vpr with host cellular proteins	35	3.19	8	0.0116	0.259
	Regulation of Glucokinase by Glucokinase Regulatory Protein	29	2.64	8	0.00345	0.161
	Rev-mediated nuclear export of HIV-1 RNA	32	2.91	8	0.00662	0.213
	Nuclear import of Rev protein	33	3	8	0.00806	0.226
	Interactions of Rev with host cellular proteins	35	3.19	8	0.0116	0.259
	Antiviral mechanism by IFN-stimulated genes	69	6.28	12	0.0206	0.298
	ISG15 antiviral mechanism	69	6.28	12	0.0206	0.298
	Glucose transport	40	3.64	8	0.0254	0.304
TCA Cycle	The citric acid (TCA) cycle and respiratory electron transport	145	13.2	24	0.00274	0.157

	Respiratory electron transport, ATP synthesis by chemiosmotic coupling, and heat production by uncoupling proteins.	101	9.2	18	0.00403	0.176
	Formation of ATP by chemiosmotic coupling	16	1.46	5	0.0115	0.259
	Regulation of pyruvate dehydrogenase (PDH) complex	13	1.18	4	0.025	0.304
Toll like receptor signaling cascade	TRIF-mediated TLR3/TLR4 signaling	87	7.92	14	0.0246	0.304
	TRAF6 Mediated Induction of proinflammatory cytokines	62	5.65	11	0.0227	0.304
	TRAF6 mediated induction of NFkB and MAP kinases upon TLR7/8 or 9 activation	76	6.92	14	0.00788	0.225
	Toll Like Receptor 7/8 (TLR7/8) Cascade	77	7.01	14	0.00885	0.226
	MyD88 dependent cascade initiated on endosome	77	7.01	14	0.00885	0.226
	Activation of the AP-1 family of transcription factors	10	0.911	4	0.00915	0.229
	Toll Like Receptor 9 (TLR9) Cascade	79	7.19	14	0.0111	0.259
	Toll Like Receptor 10 (TLR10) Cascade	74	6.74	13	0.0151	0.293
	Toll Like Receptor 5 (TLR5) Cascade	74	6.74	13	0.0151	0.293
	MyD88 cascade initiated on plasma membrane	74	6.74	13	0.0151	0.293
	MAPK targets/ Nuclear events mediated by MAP kinases	30	2.73	7	0.016	0.294
	Interleukin-1 signaling	45	4.1	9	0.0183	0.297
	eNOS activation	9	0.82	6	3.68E-05	0.0172
	Smooth Muscle Contraction	25	2.28	7	0.00561	0.213

eNOS activation pathway	Antigen Activates B Cell Receptor Leading to Generation of Second Messengers	32	2.91	7	0.0225	0.304
	Activation of CaMK IV	4	0.364	3	0.0028	0.157
	CaMK IV-mediated phosphorylation of CREB	5	0.455	3	0.00653	0.213
	Metabolism of nitric oxide	20	1.82	6	0.00709	0.213
	eNOS activation and regulation	20	1.82	6	0.00709	0.213
	CREB phosphorylation through the activation of CaMKK	6	0.546	3	0.0122	0.263
	Ionotropic activity of Kainate Receptors	12	1.09	4	0.0186	0.297
	Activation of Ca-permeable Kainate Receptor	12	1.09	4	0.0186	0.297
	Glycogen breakdown (glycogenolysis)	18	1.64	5	0.0194	0.297
	PLC beta mediated events	46	4.19	9	0.0209	0.3
	G-protein mediated events	47	4.28	9	0.0239	0.304
	Ras activation uopn Ca2+ infux through NMDA receptor	19	1.73	5	0.0244	0.304
	Tetrahydrobiopterin (BH4) synthesis, recycling, salvage and regulation	13	1.18	4	0.025	0.304
Post transcriptional modification	Regulatory RNA pathways	28	2.55	8	0.00271	0.157
	mRNA Processing	140	12.7	23	0.00367	0.166
	Processing of Capped Intron-Containing Pre-mRNA	119	10.8	19	0.0107	0.259
	mRNA Splicing	115	10.5	18	0.0155	0.293
	mRNA Splicing - Major Pathway	115	10.5	18	0.0155	0.293
	Deadenylation of mRNA	24	2.19	6	0.018	0.297
	Metabolism of RNA	339	30.9	50	0.000358	0.0836
	Cytosolic tRNA aminoacylation	24	2.19	8	0.000888	0.138

	MicroRNA (miRNA) Biogenesis	25	2.28	8	0.0012	0.153
	Metabolism of mRNA	317	28.9	44	0.00276	0.157
	Small Interfering RNA (siRNA) Biogenesis	9	0.82	4	0.00591	0.213
	mRNA Capping	30	2.73	7	0.016	0.294
	snRNP Assembly	24	2.19	6	0.018	0.297
	Metabolism of non-coding RNA	24	2.19	6	0.018	0.297

Supplemental table 4. Enriched pathways for upregulated genes in $IL8_{hi}$ at time-point 2 as compared to $IL8_{lo}$ at the same time-point.

Cluster ID	List of pathway	Total	Expected	Hits	P.Value	FDR
TLR Signaling Cascade	Cytosolic sensors of pathogen-associated DNA	19	0.962	4	0.0137	0.833
	RIP-mediated NFkB activation via DAI	11	0.557	3	0.0157	0.894
	DAI mediated induction of type I IFNs	13	0.658	4	0.0032	0.642
	Activated TLR4 signalling	100	5.06	10	0.0293	1
	MyD88-independent cascade	88	4.45	10	0.013	0.833
	Toll-Like Receptors Cascades	123	6.23	11	0.0465	1
	Toll Like Receptor 3 (TLR3) Cascade	88	4.45	10	0.013	0.833
	TRIF-mediated TLR3/TLR4 signaling	87	4.4	10	0.012	0.833
	Toll Like Receptor 4 (TLR4) Cascade	103	5.21	10	0.035	1
	TRAF6 Mediated Induction of proinflammatory cytokines	62	3.14	7	0.036	1
	Activation of the AP-1 family of transcription factors	10	0.506	3	0.0118	0.833
	MAPK targets/ Nuclear events mediated by MAP kinases	30	1.52	5	0.0162	0.894
Cytokine signaling	Signaling by Interleukins	116	5.87	13	0.00556	0.833
	Interleukin-3, 5 and GM-CSF signaling	51	2.58	8	0.00374	0.656
	Interleukin-7 signaling	12	0.607	4	0.00231	0.642
	Cytokine Signaling in Immune system	286	14.5	24	0.00978	0.833
	Inhibition of adenylate cyclase pathway	13	0.658	3	0.0252	1

Cell proliferation and growth	CREB phosphorylation through the activation of Adenylate Cyclase	5	0.253	2	0.0231	1
	PKA-mediated phosphorylation of CREB	19	0.962	4	0.0137	0.833
	PKA activation in glucagon signalling	19	0.962	4	0.0137	0.833
	Adenylate cyclase activating pathway	10	0.506	3	0.0118	0.833
	PLCG1 events in ERBB2 signaling	38	1.92	5	0.0411	1
	PLC-gamma1 signalling	37	1.87	5	0.0371	1
	PKA activation	18	0.911	4	0.0112	0.833
	Adenylate cyclase inhibitory pathway	13	0.658	3	0.0252	1
	EGFR interacts with phospholipase C-gamma	37	1.87	5	0.0371	1
	DAG and IP3 signaling	35	1.77	5	0.03	1
	Downstream signal transduction	163	8.25	14	0.0362	1
	PKA-mediated phosphorylation of key metabolic factors	6	0.304	2	0.0335	1
	Phospholipase C-mediated cascade	57	2.89	7	0.024	1
	Signaling by PDGF	189	9.57	16	0.0295	1

Supplemental table 5. Enriched pathways for downregulated genes in IL8$_{hi}$ at time-point 2 as compared to IL8$_{lo}$ at the same time-point.

Cluster ID	List of pathway	Total	Expected	Hits	P.Value	FDR
Toll-Like Receptors cascades	TRIF-mediated TLR3/TLR4 signaling	87	5.45	11	0.0192	0.718
	MyD88-independent cascade	88	5.51	11	0.0207	0.718
	Activated TLR4 signalling	100	6.26	11	0.0472	1
	Toll-Like Receptors Cascades	123	7.7	13	0.0433	1
	Toll Like Receptor 3 (TLR3) Cascade	88	5.51	11	0.0207	0.718
	Toll Like Receptor 3 (TLR3) Cascade	88	5.51	11	0.0207	0.718
Lymphocyte Activation	Adaptive Immune System	654	40.9	54	0.0191	0.718
	TCR signaling	65	4.07	8	0.0486	1
	PD-1 signaling	25	1.57	5	0.0176	0.718
	Immune System	1140	71.2	85	0.0396	1
Citric acid cycle (TCA cycle)	Citric acid cycle (TCA cycle)	26	1.63	5	0.0207	0.718
	The citric acid (TCA) cycle and respiratory electron transport	145	9.08	17	0.00881	0.686
	Respiratory electron transport, ATP synthesis by chemiosmotic coupling, and heat production by uncoupling proteins.	101	6.32	12	0.0232	0.775
	Respiratory electron transport	82	5.13	10	0.0311	0.949
Post translational protein modification	Metabolism of proteins	689	43.1	54	0.0457	1
	Post-translational protein modification	200	12.5	21	0.0132	0.718
	Synthesis of glycosylphosphatidylinositol (GPI)	17	1.06	5	0.0031	0.62
	N-glycan trimming in the ER and Calnexin/Calreticulin cycle	13	0.814	3	0.0435	1

	The activation of arylsulfatases	13	0.814	3	0.0435	1
	Post-translational modification: synthesis of GPI-anchored proteins	26	1.63	5	0.0207	0.718
	Asparagine N-linked glycosylation	86	5.38	11	0.0177	0.718
	Biosynthesis of the N-glycan precursor (dolichol lipid-linked oligosaccharide, LLO) and transfer to a nascent protein	32	2	6	0.0131	0.718
Protein folding	Chaperonin-mediated protein folding	51	3.19	9	0.00392	0.654
	Cooperation of Prefoldin and TriC/CCT in actin and tubulin folding	30	1.88	6	0.00953	0.703
	Association of TriC/CCT with target proteins during biosynthesis	29	1.82	6	0.00804	0.663
	Prefoldin mediated transfer of substrate to CCT/TriC	29	1.82	6	0.00804	0.663
	Folding of actin by CCT/TriC	9	0.563	3	0.0154	0.718
	Protein folding	56	3.51	11	0.000581	0.261

Supplemental table 6. Enriched pathways for upregulated genes at time-point 3 (T3) as compared to at time-point 1 (T1), in patients who were $IL8_{hi}$ at T1 and became $IL8_{lo}$ at T3.

Cluster ID	List of pathway	Total	Expected	Hits	P.Value	FDR
Notch signaling pathway	A third proteolytic cleavage releases NICD	9	0.148	2	0.00898	0.21
	Signaling by NOTCH1 PEST Domain Mutants in Cancer	74	1.22	5	0.00724	0.175
	Signaling by NOTCH	95	1.57	6	0.00462	0.144
	Constitutive Signaling by NOTCH1 HD+PEST Domain Mutants	52	0.857	5	0.00155	0.0661
	Signaling by NOTCH1	74	1.22	5	0.00724	0.175
	Signaling by NOTCH1 in Cancer	74	1.22	5	0.00724	0.175
	Constitutive Signaling by NOTCH1 PEST Domain Mutants	59	0.972	5	0.00273	0.0958
	Cell death signalling via NRAGE, NRIF and NADE	62	1.02	4	0.0187	0.335
	Receptor-ligand binding initiates the second proteolytic cleavage of Notch receptor	14	0.231	2	0.0215	0.355
	Signaling by NOTCH1 HD+PEST Domain Mutants in Cancer	74	1.22	5	0.00724	0.175
	Signaling by NOTCH1 t(7;9)(NOTCH1:M1580_K2555) Translocation Mutant	74	1.22	5	0.00724	0.175
	NRIF signals cell death from the nucleus	15	0.247	3	0.00171	0.0667
	Pre-NOTCH Processing in the Endoplasmic Reticulum	6	0.0988	2	0.00386	0.129

	NOTCH1 Intracellular Domain Regulates Transcription	50	0.824	4	0.00897	0.21
	Signaling by NOTCH1 HD Domain Mutants in Cancer	74	1.22	5	0.00724	0.175
	FBXW7 Mutants and NOTCH1 in Cancer	74	1.22	5	0.00724	0.175
	Activated NOTCH1 Transmits Signal to the Nucleus	29	0.478	3	0.0116	0.259
	Constitutive Signaling by NOTCH1 HD Domain Mutants	14	0.231	2	0.0215	0.355
	Signaling by NOTCH2	18	0.297	2	0.0347	0.454
	NOTCH2 Activation and Transmission of Signal to the Nucleus	18	0.297	2	0.0347	0.454
Translation of mRNA	Peptide chain elongation	178	2.93	15	0.000000175	0.0000473
	3' -UTR-mediated translational regulation	201	3.31	15	0.000000848	0.0000958
	Nonsense Mediated Decay Independent of the Exon Junction Complex	184	3.03	15	0.00000027	0.0000508
	Ribosomal scanning and start codon recognition	91	1.5	7	0.000705	0.0412
	Influenza Viral RNA Transcription and Replication	176	2.9	15	0.00000015	0.0000473
	Influenza Infection	185	3.05	15	0.00000029	0.0000508
	Influenza Life Cycle	180	2.97	15	0.000000202	0.0000473

	L13a-mediated translational silencing of Ceruloplasmin expression	201	3.31	15	0.000000848	0.0000958
	Metabolism of mRNA	317	5.22	16	0.0000546	0.00403
	GTP hydrolysis and joining of the 60S ribosomal subunit	201	3.31	15	0.000000848	0.0000958
	Eukaryotic Translation Initiation	209	3.44	15	0.00000139	0.000115
	Eukaryotic Translation Termination	178	2.93	15	0.000000175	0.0000473
	Activation of the mRNA upon binding of the cap-binding complex and eIFs, and subsequent binding to 43S	93	1.53	7	0.000804	0.0433
	Viral mRNA Translation	176	2.9	15	0.00000015	0.0000473
	Metabolism of RNA	339	5.58	16	0.000121	0.0085
	Nonsense-Mediated Decay	203	3.34	15	0.000000962	0.0000958
	Translation	249	4.1	16	0.00000261	0.000203
	Translation initiation complex formation	92	1.52	7	0.000753	0.0422
	Cap-dependent Translation Initiation	209	3.44	15	0.00000139	0.000115
	Eukaryotic Translation Elongation	186	3.06	16	4.87E-08	0.0000473
	Formation of the ternary complex, and subsequently, the 43S complex	83	1.37	7	0.000403	0.0257
	Formation of a pool of free 40S subunits	189	3.11	15	0.000000383	0.0000597

	SRP-dependent cotranslational protein targeting to membrane	204	3.36	15	0.00000102	0.0000958
	Nonsense Mediated Decay Enhanced by the Exon Junction Complex	203	3.34	15	0.000000962	0.0000958
Transcription of HIV genome	Late Phase of HIV Life Cycle	108	1.78	5	0.0324	0.454
	Formation of HIV-1 elongation complex containing HIV-1 Tat	45	0.741	3	0.0374	0.463
	Tat-mediated elongation of the HIV-1 transcript	45	0.741	3	0.0374	0.463
	Elongation arrest and recovery	33	0.544	4	0.00197	0.071
	Formation of HIV-1 elongation complex in the absence of HIV-1 Tat	46	0.758	4	0.00669	0.175
	HIV-1 elongation arrest and recovery	33	0.544	4	0.00197	0.071
	Transcription of the HIV genome	64	1.05	4	0.0208	0.355
	HIV-1 Transcription Elongation	45	0.741	3	0.0374	0.463
	Pausing and recovery of Tat-mediated HIV-1 elongation	32	0.527	3	0.0153	0.293
	RNA Polymerase II Transcription Elongation	46	0.758	4	0.00669	0.175
	Pausing and recovery of HIV-1 elongation	33	0.544	4	0.00197	0.071
	RNA Polymerase II Pre-transcription Events	62	1.02	4	0.0187	0.335
	Formation of RNA Pol II elongation complex	46	0.758	4	0.00669	0.175

	Tat-mediated HIV-1 elongation arrest and recovery	32	0.527	3	0.0153	0.293
	RNA Polymerase III Chain Elongation	18	0.297	2	0.0347	0.454
	Dual incision reaction in TC-NER	31	0.511	3	0.014	0.287
	Formation of transcription-coupled NER (TC-NER) repair complex	31	0.511	3	0.014	0.287
	RNA Polymerase III Transcription Termination	18	0.297	2	0.0347	0.454
DNA repair	DNA Repair	117	1.93	7	0.00305	0.104
	DNA strand elongation	31	0.511	3	0.014	0.287
	Processive synthesis on the C-strand of the telomere	11	0.181	2	0.0134	0.287
	Removal of the Flap Intermediate from the C-strand	10	0.165	2	0.0111	0.255
	Polymerase switching	14	0.231	3	0.00139	0.0608
	S Phase	122	2.01	6	0.0151	0.293
	Nucleotide Excision Repair	53	0.873	5	0.00169	0.0667
	Lagging Strand Synthesis	20	0.329	3	0.00404	0.132
	Telomere C-strand (Lagging Strand) Synthesis	22	0.362	3	0.00533	0.159
	Processive synthesis on the lagging strand	15	0.247	3	0.00171	0.0667
	Repair synthesis of patch ~27-30 bases long by DNA polymerase	15	0.247	2	0.0246	0.396
	Leading Strand Synthesis	14	0.231	3	0.00139	0.0608
	Gap-filling DNA repair synthesis and ligation in TC-NER	16	0.264	2	0.0278	0.423

	Polymerase switching on the C-strand of the telomere	14	0.231	3	0.00139	0.0608
	Repair synthesis for gap-filling by DNA polymerase in TC-NER	15	0.247	2	0.0246	0.396
	Removal of the Flap Intermediate from the C-strand	10	0.165	2	0.0111	0.255
	Base Excision Repair	19	0.313	2	0.0383	0.463
	Extension of Telomeres	24	0.395	3	0.00684	0.175
	G1/S-Specific Transcription	16	0.264	2	0.0278	0.423
	Resolution of AP sites via the multiple-nucleotide patch replacement pathway	17	0.28	2	0.0311	0.445
	Global Genomic NER (GG-NER)	36	0.593	3	0.0209	0.355
	Resolution of Abasic Sites (AP sites)	19	0.313	2	0.0383	0.463
	Chromosome Maintenance	124	2.04	6	0.0162	0.307
	Removal of DNA patch containing abasic residue	17	0.28	2	0.0311	0.445
	Gap-filling DNA repair synthesis and ligation in GG-NER	16	0.264	2	0.0278	0.423

Supplemental table 7. Enriched pathways for downregulated genes at time-point 3 (T3) as compared to at time-point 1 (T1), in patients who were IL8$_{hi}$ at T1 and became IL8$_{lo}$ at T3.

Cluster ID	List of pathway	Total	Expected	Hits	P.Value	FDR
TLR signaling cascade	TRAF3-dependent IRF activation pathway	14	1.69	7	0.00058	0.126
	Negative regulators of RIG-I/MDA5 signaling	33	3.98	10	0.00425	0.186
	TRAF6 mediated NF-kB activation	16	1.93	7	0.00155	0.128
	NF-kB activation through FADD/RIP-1 pathway mediated by caspase-8 and -10	12	1.45	6	0.00147	0.128
	RIG-I/MDA5 mediated induction of IFN-alpha/beta pathways	67	8.09	17	0.00199	0.139
	Cytosolic sensors of pathogen-associated DNA	19	2.29	7	0.00494	0.197
	RIP-mediated NFkB activation via DAI	11	1.33	5	0.00626	0.204
	DAI mediated induction of type I IFNs	13	1.57	7	0.000323	0.126
	Toll Like Receptor 3 (TLR3) Cascade	88	10.6	20	0.00346	0.167
	Toll Like Receptor 4 (TLR4) Cascade	103	12.4	23	0.00228	0.139
	MyD88-independent cascade	88	10.6	20	0.00346	0.167
	TRIF-mediated TLR3/TLR4 signaling	87	10.5	20	0.003	0.156
	Activated TLR4 signalling	100	12.1	21	0.00734	0.219
	Toll-Like Receptors Cascades	123	14.8	25	0.00567	0.197

	TRAF6 Mediated Induction of proinflammatory cytokines	62	7.48	13	0.0314	0.373
Transcription and Post Transcriptional modification	mRNA Processing	140	16.9	30	0.00108	0.128
	mRNA Splicing - Major Pathway	115	13.9	25	0.00222	0.139
	RNA Polymerase II Pre-transcription Events	62	7.48	13	0.0314	0.373
	HIV-1 Transcription Initiation	41	4.95	10	0.0212	0.337
	RNA Polymerase II Transcription Pre-Initiation And Promoter Opening	41	4.95	10	0.0212	0.337
	RNA Polymerase II Transcription	107	12.9	21	0.0158	0.33
	Processing of Capped Intron-Containing Pre-mRNA	119	14.4	25	0.00361	0.169
	mRNA Splicing	115	13.9	25	0.00222	0.139
	RNA Polymerase II Transcription Initiation And Promoter Clearance	41	4.95	10	0.0212	0.337
	RNA Polymerase II Promoter Escape	41	4.95	10	0.0212	0.337
	RNA Polymerase II Transcription Initiation	41	4.95	10	0.0212	0.337
	Transcription	149	18	31	0.0015	0.128
	RNA Polymerase II HIV-1 Promoter Escape	41	4.95	10	0.0212	0.337
mTOR pathway	S6K1-mediated signalling	9	1.09	4	0.0161	0.33
	Calnexin/calreticulin cycle	11	1.33	5	0.00626	0.204

	ER Quality Control Compartment (ERQC)	6	0.724	3	0.0265	0.357
	N-glycan trimming in the ER and Calnexin/Calreticulin cycle	13	1.57	5	0.0142	0.33
	PKB-mediated events	30	3.62	9	0.00708	0.216
	mTOR signalling	29	3.5	9	0.00553	0.197
	Regulation of Rheb GTPase activity by AMPK	10	1.21	4	0.0243	0.348
Cytokine Signaling	CD28 co-stimulation	30	3.62	9	0.00708	0.216
	Costimulation by the CD28 family	68	8.21	14	0.0302	0.371
	CD28 dependent PI3K/Akt signaling	19	2.29	6	0.0206	0.337
	Antigen Activates B Cell Receptor Leading to Generation of Second Messengers	32	3.86	11	0.000865	0.128
	Innate Immune System	521	62.9	81	0.00822	0.226
	Fcgamma receptor (FCGR) dependent phagocytosis	86	10.4	21	0.00106	0.128
	Regulation of actin dynamics for phagocytic cup formation	62	7.48	13	0.0314	0.373
	Signaling by Interleukins	116	14	22	0.02	0.337
	Cytokine Signaling in Immune system	286	34.5	50	0.00393	0.178
Lymphocyte Activation	Class I MHC mediated antigen processing & presentation	267	32.2	49	0.00154	0.128
	Antigen processing: Ubiquitination & Proteasome degradation	224	27	44	0.000631	0.126
	Adaptive Immune System	654	78.9	103	0.00194	0.139

	Signaling by the B Cell Receptor (BCR)	199	24	38	0.00246	0.143
Cell cycle checkpoint	Ubiquitin-dependent degradation of Cyclin D	49	5.91	11	0.0289	0.369
	p53-Independent G1/S DNA damage checkpoint	52	6.28	12	0.0187	0.337
	Host Interactions of HIV factors	141	17	26	0.0172	0.337
	Vif-mediated degradation of APOBEC3G	55	6.64	12	0.0285	0.369
	CDK-mediated phosphorylation and removal of Cdc6	49	5.91	11	0.0289	0.369
	Regulation of mRNA Stability by Proteins that Bind AU-rich Elements	88	10.6	21	0.00146	0.128
	Degradation of beta-catenin by the destruction complex	65	7.85	14	0.021	0.337
	Cyclin E associated events during G1/S transition	65	7.85	14	0.021	0.337
	Autodegradation of Cdh1 by Cdh1:APC/C	68	8.21	14	0.0302	0.371
	Ubiquitin-dependent degradation of Cyclin D1	49	5.91	11	0.0289	0.369
	Stabilization of p53	54	6.52	12	0.0249	0.348
	Autodegradation of the E3 ubiquitin ligase COP1	53	6.4	12	0.0217	0.337
	Cross-presentation of soluble exogenous antigens (endosomes)	48	5.79	12	0.00992	0.263
	Activation of NF-kappaB in B Cells	66	7.97	14	0.0238	0.348

	Cyclin A:Cdk2-associated events at S phase entry	66	7.97	14	0.0238	0.348
	p53-Independent DNA Damage Response	52	6.28	12	0.0187	0.337
	Destabilization of mRNA by AUF1 (hnRNP D0)	54	6.52	12	0.0249	0.348
	Regulation of activated PAK-2p34 by proteasome mediated degradation	48	5.79	11	0.0251	0.348
	Signaling by Wnt	65	7.85	14	0.021	0.337
	Ubiquitin Mediated Degradation of Phosphorylated Cdc25A	52	6.28	12	0.0187	0.337

Supplemental code 1. Code for Bayesian information criterion analysis

```
> data = read.csv(choose.files(), header = TRUE)
> new_data = data[,-1]
## Stepwise regression using BIC
> library(MASS)
> full.model<- lm(AUC ~ ., data=new_data)
> summary(full.model)
> step.model<- stepAIC(full.model, k = log(nrow(new_data)), direction = "both", trace = FALSE)
  # Use k = 2 for AIC, log(n) for BIC
> step.model$coefficients
> summary(step.model)
> step.model<- stepAIC(full.model, k = 2, direction = "both", trace = FALSE) # Use k = 2 for AIC,
  log(n) for BIC
> step.model$coefficients
> summary(step.model)
## plot for bic
> X = matrix(0, nrow = 37, ncol = 1000)
> AIC_vec = c()
> Rsq_vec = c()
> adj.Rsq_vec = c()
> current.model = full.model
> for(i in 1:1000){
  AIC_vec[i] = extractAIC(current.model, k = log(nrow(new_data)))[2]
  Rsq_vec[i] = summary(current.model)$r.squared
  adj.Rsq_vec[i] = summary(current.model)$adj.r.squared
  X[,i] = (names(full.model$coefficients) %in% names(current.model$coefficients))*1
  new.model<- stepAIC(current.model, k = log(nrow(new_data)), direction = "both", steps = 1,
  trace = FALSE)
  if(all(names(new.model$coefficients)%in% names(current.model$coefficients))
  && all(names(current.model$coefficients)%in% names(new.model$coefficients))){
  break
  } else{
  current.model = new.model
  }
```

```
}
>    conv = length(AIC_vec)
>    X = X[, 1:conv]
## variable plot
>    library(reshape2)
>    library(ggplot2)
>    longData<-melt(X)
>    longData<-longData[longData$value!=0,]
>    longData[,3]= as.factor(longData[,3])
>    p5 <- ggplot(longData, aes(x = Var2, y = Var1)) +
     geom_raster(,fill="grey40") +
     labs(x="Iteration", y="Variables", title="Variables selected") +
     theme_bw() +
     scale_y_discrete(limits = names(full.model$coefficients))
>    p5
## bic plot
>    xValue<- 1:conv
>    data <- data.frame(xValue,AIC_vec, Rsq_vec, adj.Rsq_vec)
>    p6 <- ggplot(data, aes(x=xValue)) +
     geom_line(aes(y = AIC_vec, col = "BIC"), lwd = 1.2) +
     geom_point(aes(y = AIC_vec, col = "BIC"), pch = 16, size= 2) +
     theme_bw() +
     labs(x="Iteration", y="BIC", title="Variation of BIC")
>    p6
## R^2 plot
>    p7 <- ggplot(data, aes(x=xValue)) +
     geom_line(aes(y = Rsq_vec, col = "R squared"), lwd = 1.2) +
     geom_point(aes(y = Rsq_vec, col = "R squared"), pch = 16, size= 2) +
     geom_line(aes(y = adj.Rsq_vec, col = "Adjusted R squared"), lwd = 1.2) +
     geom_point(aes(y = adj.Rsq_vec, col = "Adjusted R squared"), pch = 16, size= 2) +
     theme_bw() +
     labs(x="Iteration", y=" ", title="Variation of R squared and adjusted R squared")
>    p7
## panel plot
```

```
> library(ggpubr)
> p8<- ggarrange(p5, ggarrange(p6, p7, ncol = 1), nrow = 1)
> p8 <- annotate_figure(p8, top = text_grob("Stepwise Regression using BIC", face = "bold", size
  = 14))
> p8
> ggsave("variable_selection_bic.jpg", p8, width = 12, height = 10, units = "in")
```

Supplemental code 2. R codes for receiver operator characteristics analysis. Code shown is for analysis with IL-8 data. Same code was used for other analyses also.

```
> rm(list=ls())
> library(pROC)
> library(randomForest)
> IL8_data <- read.csv(choose.files())
> plot(x=IL8_data$IL.8, y=IL8_data$Death)
> glm.fit = glm(IL8_data$Death ~ IL8_data$IL.8, family = binomial)
> lines(IL8_data$IL.8, glm.fit$fitted.values)
> par(pty = 's')
> roc(IL8_data$Death, glm.fit$fitted.values, plot=TRUE,legacy.axes= TRUE)
> roc.info <- roc(IL8_data$Death, glm.fit$fitted.values, legacy.axes = TRUE)
> roc.df<- data.frame(tpp=roc.info$sensitivities*100, fpp=(1-roc.info$sensitivities)*100,
  thresholds=roc.info$thresholds)
> library(cutpointr)
> cp <- cutpointr(IL8_data, IL.8, Death,  method = maximize_metric, metric = sum_sens_spec)
> summary(cp)
> plot(cp)
```

Supplemental code 3. R codes for analyses of single cell RNA sequencing dataset GSE163668 (mild COVID-19 patients).

```
> rm(list=ls())
> library(Seurat)
> library(Matrix)
> library(dplyr)
> data_dir<- 'D:\\DC lab\\IL8_ANALYSIS\\GSE163668 POOLED\\GSE163668 POOLED 5,6 MILD'
> expression_matrix<- Read10X(data.dir = data_dir)
```

```
> sample.tmp.seurat<- CreateSeuratObject(counts = expression_matrix, min.cells = 3, min.features = 200)
> sample.tmp.seurat<- NormalizeData(sample.tmp.seurat)
> sample.tmp.seurat<- FindVariableFeatures(sample.tmp.seurat, selection.method = "vst", nfeatures = 4000)
> nCoV.integrated<- ScaleData(sample.tmp.seurat)
> nCoV.integrated<- RunPCA(nCoV.integrated, verbose = FALSE,npcs = 100)
> nCoV.integrated<- ProjectDim(object = nCoV.integrated)
> nCoV.integrated<- FindNeighbors(object = nCoV.integrated, dims = 1:50)
> nCoV.integrated<- FindClusters(object = nCoV.integrated, resolution = 1.2)
> nCoV.integrated<- RunTSNE(object = nCoV.integrated, dims = 1:50)
> DimPlot(object = nCoV.integrated, reduction = 'tsne',label = TRUE)
> FeaturePlot(nCoV.integrated,features=c("CXCL8", "CXCR1"), cols=c("Dark Grey", "Red"))
```

Supplemental code 4. R codes for analyses of single cell RNA sequencing dataset GSE163668 (severe COVID-19 patients).

```
> rm(list=ls())
> library(Seurat)
> library(Matrix)
> library(dplyr)
> data_dir<- 'D:\\DC lab\\IL8_ANALYSIS\\GSE163668 POOLED\\GSE163668 POOLED 1,2,3 SEVERE'
> expression_matrix<- Read10X(data.dir = data_dir)
> sample.tmp.seurat<- CreateSeuratObject(counts = expression_matrix, min.cells = 3, min.features = 200)
> sample.tmp.seurat<- NormalizeData(sample.tmp.seurat)
> sample.tmp.seurat<- FindVariableFeatures(sample.tmp.seurat, selection.method = "vst", nfeatures = 4000)
> nCoV.integrated<- ScaleData(sample.tmp.seurat)
> nCoV.integrated<- RunPCA(nCoV.integrated, verbose = FALSE,npcs = 100)
> nCoV.integrated<- ProjectDim(object = nCoV.integrated)
> nCoV.integrated<- FindNeighbors(object = nCoV.integrated, dims = 1:50)
> nCoV.integrated<- FindClusters(object = nCoV.integrated, resolution = 1.2)
> nCoV.integrated<- RunTSNE(object = nCoV.integrated, dims = 1:50)
> DimPlot(object = nCoV.integrated, reduction = 'tsne',label = TRUE)
> FeaturePlot(nCoV.integrated,features=c("CXCL8", "CXCR1"), cols=c("Dark Grey", "Red"))
```

```
>
> CD3_expression = GetAssayData(object = nCoV.integrated)["CD3E",]
> VlnPlot(object = nCoV.integrated, features = c("CD3E"),pt.size = 0)
> CD3_ids = names(which(CD3_expression>1))
> CD3_cells = subset(nCoV.integrated,cells=CD3_ids)
> CD4_expression = GetAssayData(object = nCoV.integrated)["CD4",]
> VlnPlot(object = nCoV.integrated, features = c("CD4"),pt.size = 0)
> CD4_ids2 = names(which(CD4_expression>0.3))
> CD3_CD4_cells = subset(CD3_cells,cells=CD4_ids2)
> FeaturePlot(CD3_CD4_cells,features=c("CXCL8", "CXCR1"), cols=c("Dark Grey", "Red"))
> CD8_expression = GetAssayData(object = nCoV.integrated)["CD8B",]
> VlnPlot(object = nCoV.integrated, features = c("CD4"),pt.size = 0)
> CD8_ids = names(which(CD8_expression>0.5))
> CD3_CD8_cells = subset(CD3_cells,cells=CD8_ids)
> FeaturePlot(CD3_CD8_cells,features=c("CXCL8", "CXCR1"), cols=c("Dark Grey", "Red"))
> CD14_expression = GetAssayData(object = nCoV.integrated)["CD14",]
> VlnPlot(object = nCoV.integrated, features = c("CD14"),pt.size = 0)
> CD14_ids = names(which(CD3_expression>1))
> CD14_ids = names(which(CD14_expression>1))
> CD14_cells = subset(nCoV.integrated,cells=CD14_ids)
> FeaturePlot(CD14_cells,features=c("CXCL8", "CXCR1"), cols=c("Dark Grey", "Red"))
> CD19_expression = GetAssayData(object = nCoV.integrated)["CD19",]
> VlnPlot(object = nCoV.integrated, features = c("CD19"),pt.size = 0)
> CD19_ids = names(which(CD19_expression>0.5))
> CD19_cells = subset(nCoV.integrated,cells=CD19_ids)
> FeaturePlot(CD19_cells,features=c("CXCL8", "CXCR1"), cols=c("Dark Grey", "Red"))
> VlnPlot(object = nCoV.integrated, features = c("NCAM1"),pt.size = 0)
> NCAM1_expression = GetAssayData(object = nCoV.integrated)["NCAM1",]
> NCAM1_ids = names(which(NCAM1_expression>0.5))
> NCAM1_cells = subset(nCoV.integrated,cells=NCAM1_ids)
> FeaturePlot(NCAM1_cells,features=c("CXCL8", "CXCR1"), cols=c("Dark Grey", "Red"))
> VlnPlot(object = nCoV.integrated, features = c("FCGR3A"),pt.size = 0)
> FCGR3A_expression = GetAssayData(object = nCoV.integrated)["FCGR3A",]
> FCGR3A_ids = names(which(FCGR3A_expression>1))
```

```
> FCGR3A_cells = subset(nCoV.integrated,cells=FCGR3A_ids)
> FeaturePlot(FCGR3A_cells,features=c("CXCL8", "CXCR1"), cols=c("Dark Grey", "Red"))
> VlnPlot(object = nCoV.integrated, features = c("ITGAM"),pt.size = 0)
> ITGAM_expression = GetAssayData(object = nCoV.integrated)["ITGAM",]
> ITGAM_ids = names(which(ITGAM_expression>0.5))
> ITGAM_cells = subset(nCoV.integrated,cells=ITGAM_ids)
> FeaturePlot(ITGAM_cells,features=c("CXCL8", "CXCR1"), cols=c("Dark Grey", "Red"))
> ITGAX_expression = GetAssayData(object = nCoV.integrated)["ITGAX",]
> VlnPlot(object = nCoV.integrated, features = c("ITGAX"),pt.size = 0)
> ITGAX_ids = names(which(ITGAX_expression>0.5))
> ITGAX_cells = subset(nCoV.integrated,cells=ITGAX_ids)
> FeaturePlot(ITGAX_cells,features=c("CXCL8", "CXCR1"), cols=c("Dark Grey", "Red"))
```

Supplemental code 5. R codes for analyses of single cell RNA sequencing dataset GSE145926 (mild COVID-19 patients).

```
>	rm(list=ls())
>	library(Seurat)
>	library(Matrix)
>	library(dplyr)
>	expression_matrix1 <- Read10X_h5(choose.files())
>	sample.tmp.seurat1 <- CreateSeuratObject(counts = expression_matrix1, min.cells = 3, 
min.features = 200)
>	expression_matrix2 <- Read10X_h5(choose.files())
>	sample.tmp.seurat2 <- CreateSeuratObject(counts = expression_matrix2, min.cells = 3, 
min.features = 200)
>	sample.tmp.seurat.combined<- merge(sample.tmp.seurat1, y = sample.tmp.seurat2)
>	expression_matrix3 <- Read10X_h5(choose.files())
>	sample.tmp.seurat3 <- CreateSeuratObject(counts = expression_matrix3, min.cells = 3, 
min.features = 200)
>	sample.tmp.seurat.combined.mild<- merge(sample.tmp.seurat.combined, y = 
sample.tmp.seurat3)
>	sample.tmp.seurat.combined.mild<- NormalizeData(sample.tmp.seurat.combined.mild, 
verbose = FALSE)
```

```
>	sample.tmp.seurat.combined.mild<-
FindVariableFeatures(sample.tmp.seurat.combined.mild, selection.method = "vst", nfeatures =
2000,verbose = FALSE)
>	nCoV.integrated<- ScaleData(sample.tmp.seurat.combined.mild, verbose = FALSE)
>	nCoV.integrated<- RunPCA(nCoV.integrated, verbose = FALSE,npcs = 100)
>	nCoV.integrated.mild<- ProjectDim(object = nCoV.integrated)
>	nCoV.integrated<- FindNeighbors(object = nCoV.integrated, dims = 1:50)
>	nCoV.integrated<- FindClusters(object = nCoV.integrated, resolution = 0.5)
>	nCoV.integrated<- RunTSNE(object = nCoV.integrated, dims = 1:50)
>	DimPlot(object = nCoV.integrated, reduction = 'tsne',label = TRUE)
>	FeaturePlot(nCoV.integrated,features=c("CXCL8", "CXCR1"), cols=c("light Grey", "Red"))
```

Supplemental code 6. R codes for analyses of single cell RNA sequencing dataset GSE145926 (severe COVID-19 patients).

```
> rm(list=ls())
> library(Seurat)
> library(Matrix)
> library(dplyr)
> expression_matrix1 <- Read10X_h5(choose.files())
> sample.tmp.seurat1 <- CreateSeuratObject(counts = expression_matrix1, min.cells = 3,
  min.features = 200)
> expression_matrix2 <- Read10X_h5(choose.files())
> sample.tmp.seurat2 <- CreateSeuratObject(counts = expression_matrix2, min.cells = 3,
  min.features = 200)
> sample.tmp.seurat.combined<- merge(sample.tmp.seurat1, y = sample.tmp.seurat2)
> expression_matrix3 <- Read10X_h5(choose.files())
> sample.tmp.seurat3 <- CreateSeuratObject(counts = expression_matrix3, min.cells = 3,
  min.features = 200)
> sample.tmp.seurat.combined2<- merge(sample.tmp.seurat.combined, y = sample.tmp.seurat3)
> expression_matrix4 <- Read10X_h5(choose.files())
> sample.tmp.seurat4 <- CreateSeuratObject(counts = expression_matrix4, min.cells = 3,
  min.features = 200)
> sample.tmp.seurat.combined3<- merge(sample.tmp.seurat.combined2, y = sample.tmp.seurat4)
> expression_matrix5 <- Read10X_h5(choose.files())
```

```
> sample.tmp.seurat5 <- CreateSeuratObject(counts = expression_matrix5, min.cells = 3, min.features = 200)
> sample.tmp.seurat.combined4<- merge(sample.tmp.seurat.combined3, y = sample.tmp.seurat5)
> expression_matrix6 <- Read10X_h5(choose.files())
> sample.tmp.seurat6 <- CreateSeuratObject(counts = expression_matrix6, min.cells = 3, min.features = 200)
> sample.tmp.seurat.combined5<- merge(sample.tmp.seurat.combined4, y = sample.tmp.seurat6)
> sample.tmp.seurat.combined5<- NormalizeData(sample.tmp.seurat.combined5, verbose = FALSE)
> sample.tmp.seurat.combined5<- FindVariableFeatures(sample.tmp.seurat.combined5, selection.method = "vst", nfeatures = 2000,verbose = FALSE)
> nCoV.integrated.severe<- ScaleData(sample.tmp.seurat.combined5, verbose = FALSE)
> nCoV.integrated.severe<- RunPCA(nCoV.integrated.severe, verbose = FALSE,npcs = 100)
> nCoV.integrated.severe.final<- ProjectDim(object = nCoV.integrated.severe)
> nCoV.integrated.severe<- FindNeighbors(object = nCoV.integrated.severe, dims = 1:50)
> nCoV.integrated.severe<- FindClusters(object = nCoV.integrated.severe, resolution = 0.5)
> nCoV.integrated.severe<- RunTSNE(object = nCoV.integrated.severe, dims = 1:50)
> DimPlot(object = nCoV.integrated.severe, reduction = 'tsne',label = TRUE)
> FeaturePlot(nCoV.integrated.severe,features=c("CXCL8", "CXCR1"), cols=c("light Grey", "Red"))
> FeaturePlot(nCoV.integrated.severe,"EPCAM", cols=c("Dark Grey", "Blue"))
> FeaturePlot(nCoV.integrated.severe,"CXCL8", cols=c("Dark Grey", "Blue"))
> FeaturePlot(nCoV.integrated.severe,"CD4", cols=c("Dark Grey", "Blue"))
> FeaturePlot(nCoV.integrated.severe,"CD8B", cols=c("Dark Grey", "Blue"))
> FeaturePlot(nCoV.integrated.severe,"CD19", cols=c("Dark Grey", "Blue"))
> FeaturePlot(nCoV.integrated.severe,"NCAM1", cols=c("Dark Grey", "Blue"))
> FeaturePlot(nCoV.integrated.severe,"CD14", cols=c("Dark Grey", "Blue"))
> FeaturePlot(nCoV.integrated.severe,"FCGR3A", cols=c("Dark Grey", "Blue"))
> FeaturePlot(nCoV.integrated.severe,"ITGAM", cols=c("Dark Grey", "Blue"))
> FeaturePlot(nCoV.integrated.severe,"ITGAX", cols=c("Dark Grey", "Blue"))
> EPCAM_expression = GetAssayData(object = nCoV.integrated.severe)["EPCAM",]
> CD4_expression = GetAssayData(object = nCoV.integrated.severe)["CD4",]
> CD8B_expression = GetAssayData(object = nCoV.integrated.severe)["CD8B",]
> CD19_expression = GetAssayData(object = nCoV.integrated.severe)["CD19",]
> NCAM1_expression = GetAssayData(object = nCoV.integrated.severe)["NCAM1",]
> CD14_expression = GetAssayData(object = nCoV.integrated.severe)["CD14",]
```

```
> FCGR3A_expression = GetAssayData(object = nCoV.integrated.severe)["FCGR3A",]
> ITGAM_expression = GetAssayData(object = nCoV.integrated.severe)["ITGAM",]
> ITGAX_expression = GetAssayData(object = nCoV.integrated.severe)["ITGAX",]
> CD3_expression = GetAssayData(object = nCoV.integrated.severe)["CD3E",]
> CD3_ids = names(which(CD3_expression>0.5))
> CD3_cells = subset(nCoV.integrated.severe,cells=CD3_ids)
> FeaturePlot(CD3_cells,"CXCL8")
> CD4_expression = GetAssayData(object = nCoV.integrated.severe)["CD4",]
> VlnPlot(object = nCoV.integrated.severe, features = c("CD4"),pt.size = 0)
> CD4_ids = names(which(CD4_expression>0.2))
> CD4_cells = subset(nCoV.integrated.severe,cells=CD4_ids)
> CD4_cells = subset(CD3_cells,cells=CD4_ids)
> FeaturePlot(CD4_cells,"CXCL8",cols=c("Dark Grey", "Red"))
> FeaturePlot(CD4_cells,"CXCR1",cols=c("Dark Grey", "Red"))
> CD8_ids = names(which(CD8B_expression>0.5))
> CD8_cells = subset(CD3_cells,cells=CD8_ids)
> FeaturePlot(CD8_cells,"CXCL8",cols=c("Dark Grey", "Red"))
> FeaturePlot(CD8_cells,"CXCR1",cols=c("Dark Grey", "Red"))
> CD19_ids = names(which(CD19_expression>0.2))
> CD19_cells = subset(nCoV.integrated.severe,cells=CD19_ids)
> FeaturePlot(CD19_cells,"CXCL8",cols=c("Dark Grey", "Red"))
> FeaturePlot(CD19_cells,"CXCR1",cols=c("Dark Grey", "Red"))
> VlnPlot(object = nCoV.integrated.severe, features = c("NCAM1"),pt.size = 0)
> NCAM1_ids = names(which(NCAM1_expression>0.5))
> NCAM1_cells = subset(nCoV.integrated.severe,cells=NCAM1_ids)
> FeaturePlot(NCAM1_cells,"CXCL8",cols=c("Dark Grey", "Red"))
> FeaturePlot(NCAM1_cells,"CXCR1",cols=c("Dark Grey", "Red"))
> VlnPlot(object = nCoV.integrated.severe, features = c("CD14"),pt.size = 0)
> CD14_ids = names(which(CD14_expression>0.3))
> CD14_cells = subset(nCoV.integrated.severe,cells=CD14_ids)
> FeaturePlot(CD14_cells,"CXCL8", cols=c("Dark Grey", "Red"))
> FeaturePlot(CD14_cells,"CXCR1", cols=c("Dark Grey", "Red"))
> VlnPlot(object = nCoV.integrated.severe, features = c("FCGR3A"),pt.size = 0)
> FCGR3A_ids = names(which(FCGR3A_expression>0.3))
```

```
> FCGR3A_cells = subset(nCoV.integrated.severe,cells=FCGR3A_ids)
> FeaturePlot(FCGR3A_cells,"CXCL8", cols=c("Dark Grey", "Red"))
> FeaturePlot(FCGR3A_cells,"CXCR1", cols=c("Dark Grey", "Red"))
> VlnPlot(object = nCoV.integrated.severe, features = c("ITGAM"),pt.size = 0)
> ITGAM_ids = names(which(ITGAM_expression>0.2))
> ITGAM_cells = subset(nCoV.integrated.severe,cells=ITGAM_ids)
> FeaturePlot(ITGAM_cells,"CXCL8", cols=c("Dark Grey", "Red"))
> FeaturePlot(ITGAM_cells,"CXCR1", cols=c("Dark Grey", "Red"))
> VlnPlot(object = nCoV.integrated.severe, features = c("ITGAX"),pt.size = 0)
> ITGAX_ids = names(which(ITGAX_expression>0.3))
> ITGAX_cells = subset(nCoV.integrated.severe,cells=ITGAX_ids)
> FeaturePlot(ITGAX_cells,"CXCL8", cols=c("Dark Grey", "Red"))
> FeaturePlot(ITGAX_cells,"CXCR1", cols=c("Dark Grey", "Red"))
```
